# Supplementary figures and images for: A new root-knot nematode, Meloidogyne vitis sp. nov. (Nematoda: Meloidogynidae), parasitizing grape in Yunnan
Source: PLoS One. 2021 Feb 3;16(2):e0245201. doi: 10.1371/journal.pone.0245201 (PMC7857618; doi:10.1371/journal.pone.0245201)

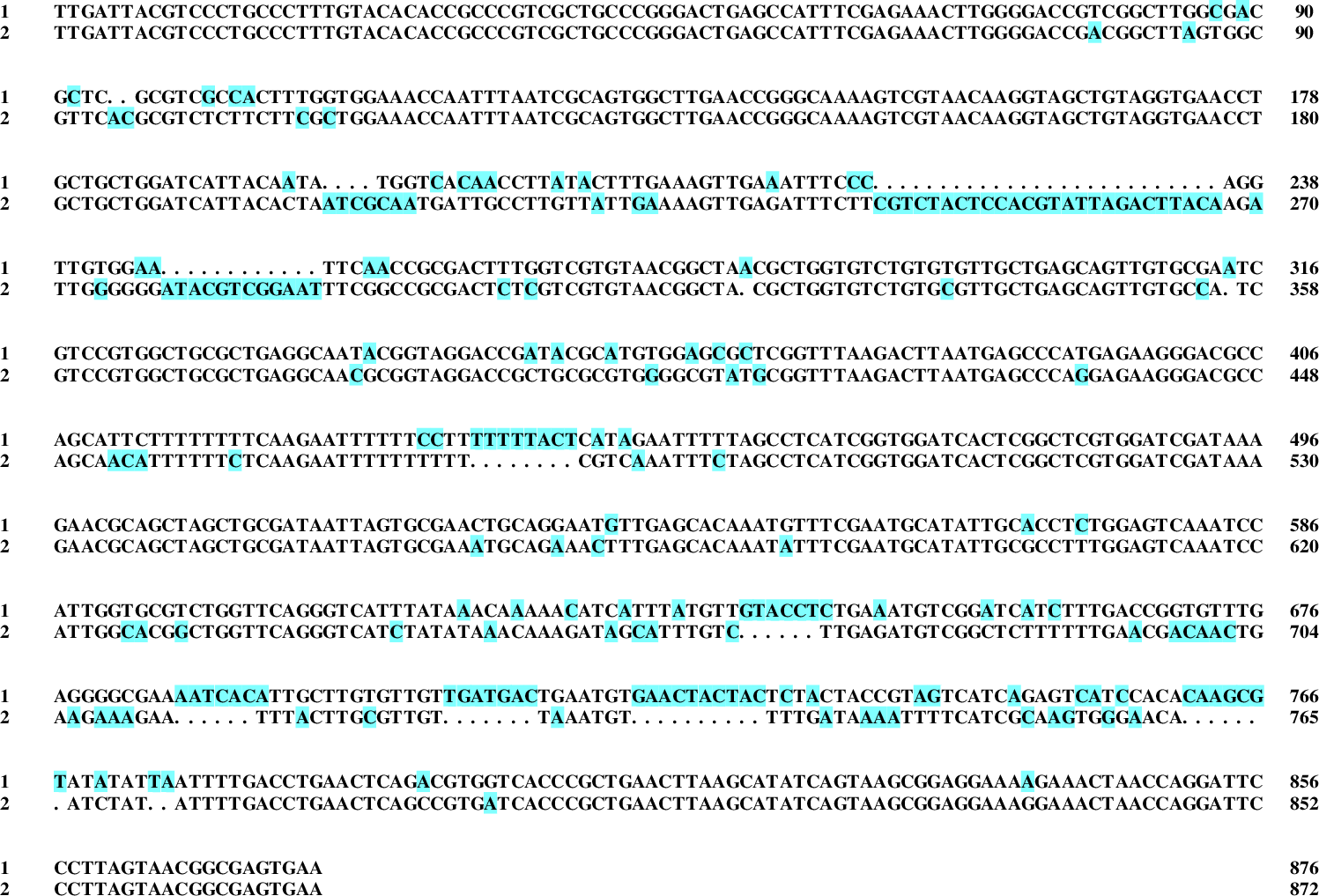

Supplement: S1 Fig — (1 = Meloidogyne vitis sp. nov., 2 = Meloidogyne mali). (TIF) [file pone.0245201.s001.tif]

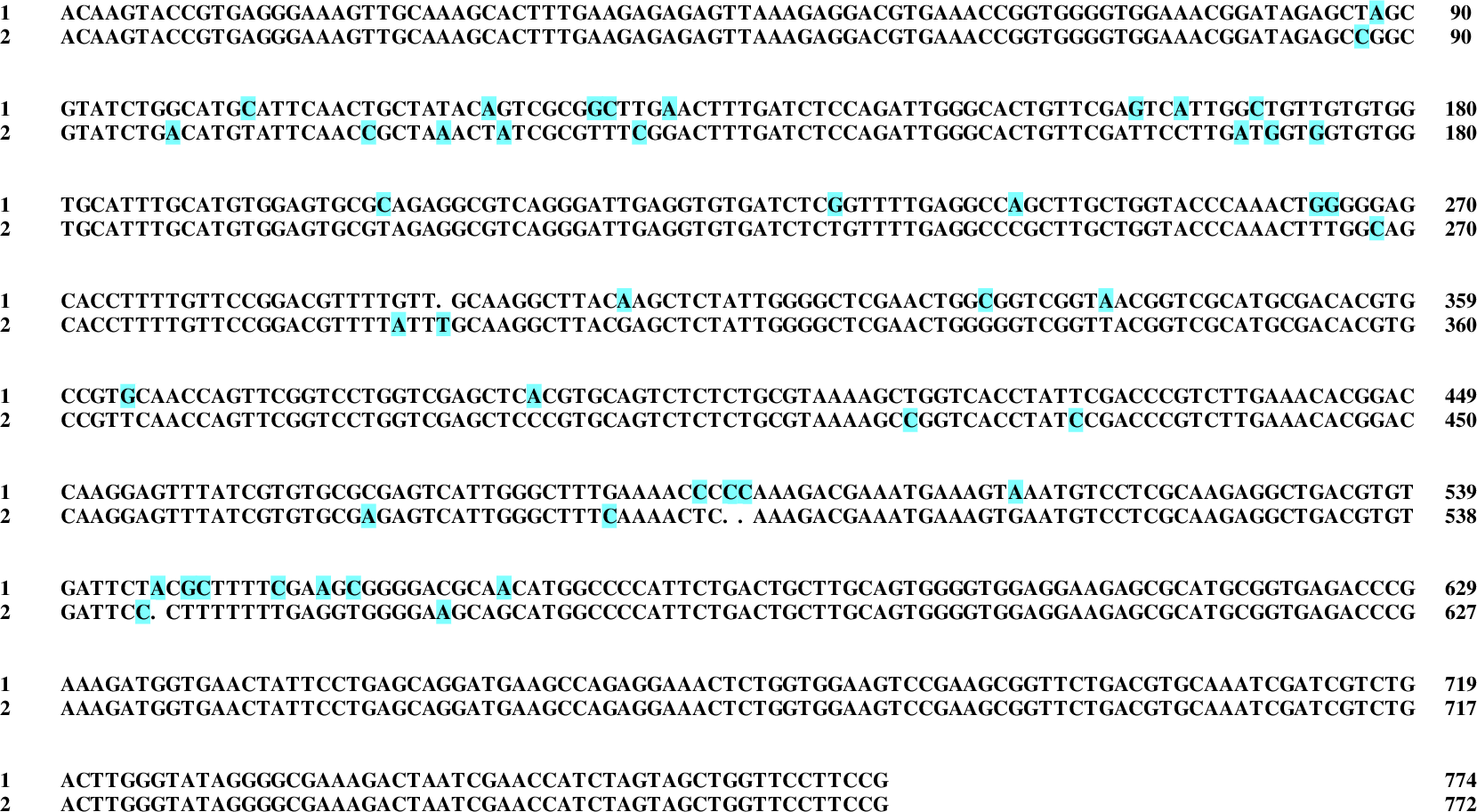

Supplement: S2 Fig — (1 = Meloidogyne vitis sp. nov., 2 = Meloidogyne mali). (TIF) [file pone.0245201.s002.tif]

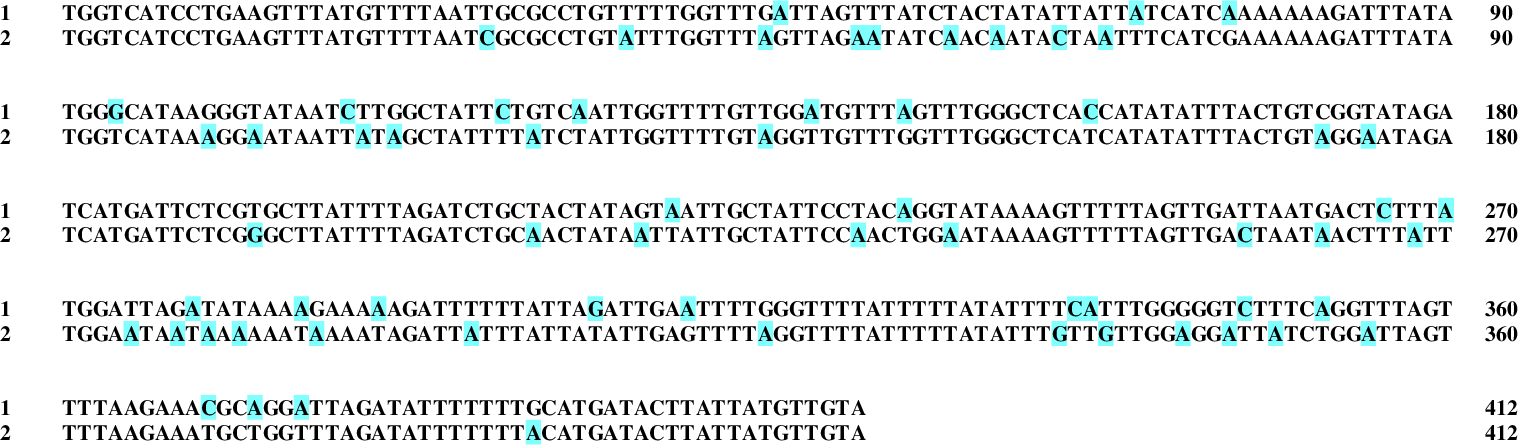

Supplement: S3 Fig — (1 = Meloidogyne vitis sp. nov., 2 = Meloidogyne mali). (TIF) [file pone.0245201.s003.tif]

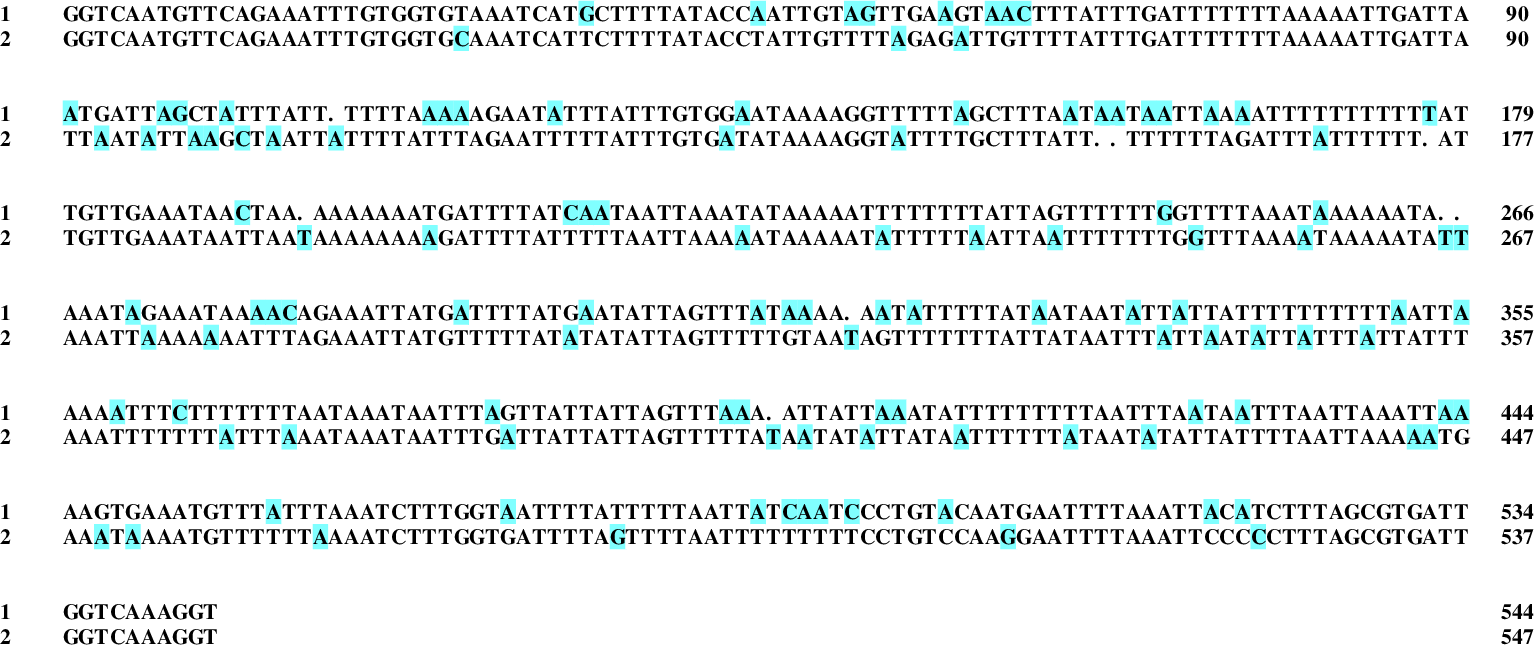

Supplement: S4 Fig — (1 = Meloidogyne vitis sp. nov., 2 = Meloidogyne mali). (TIF) [file pone.0245201.s004.tif]
