# Supplementary material for: A new root-knot nematode, Meloidogyne vitis sp. nov. (Nematoda: Meloidogynidae), parasitizing grape in Yunnan
Source: PLoS One. 2021 Feb 3;16(2):e0245201. doi: 10.1371/journal.pone.0245201 (PMC7857618; doi:10.1371/journal.pone.0245201)
Supplement: S1 Raw images — (PDF) [file pone.0245201.s005.pdf]

Original images

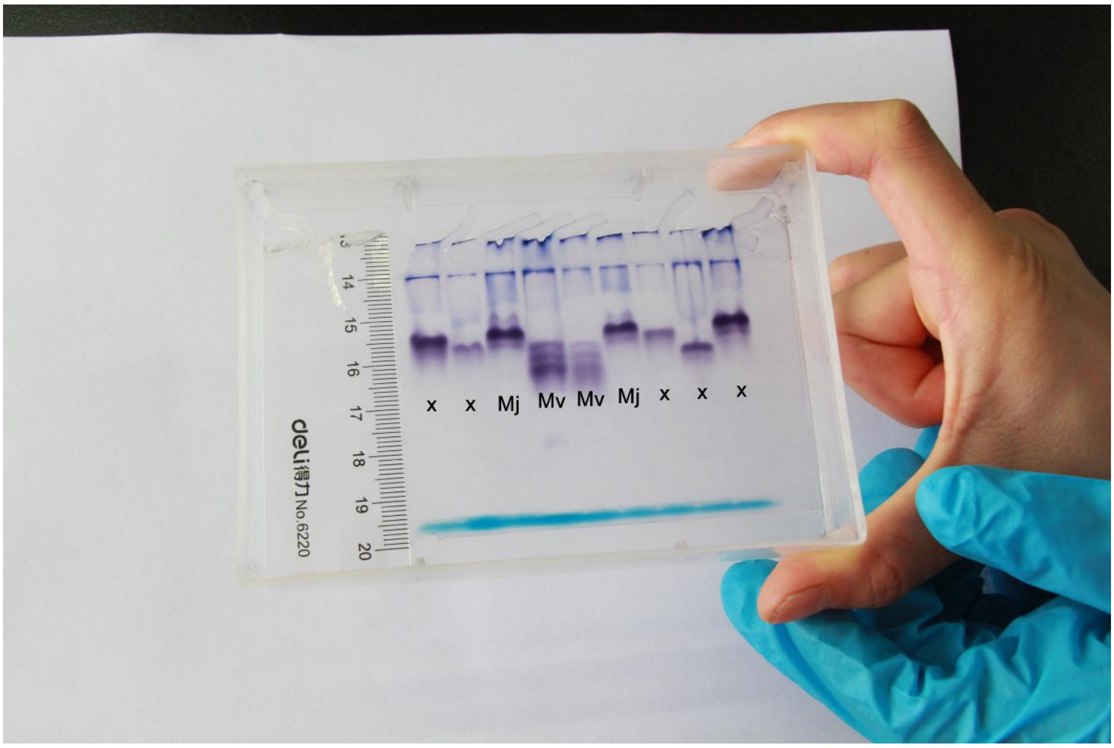

Fig 5-A: Malate dehydrogenase phenotype patterns obtained with electrophoresis of protein homogenates from five young egg-laying females of *Meloidogyne vitis* sp. nov. (lane Mv) and five young egg-laying females of the *Meloidogyne javanica* reference population (lane Mj). Lanes "X": lanes not included in the final figure.

The image was captured from iPhone 6 A1699 (America).

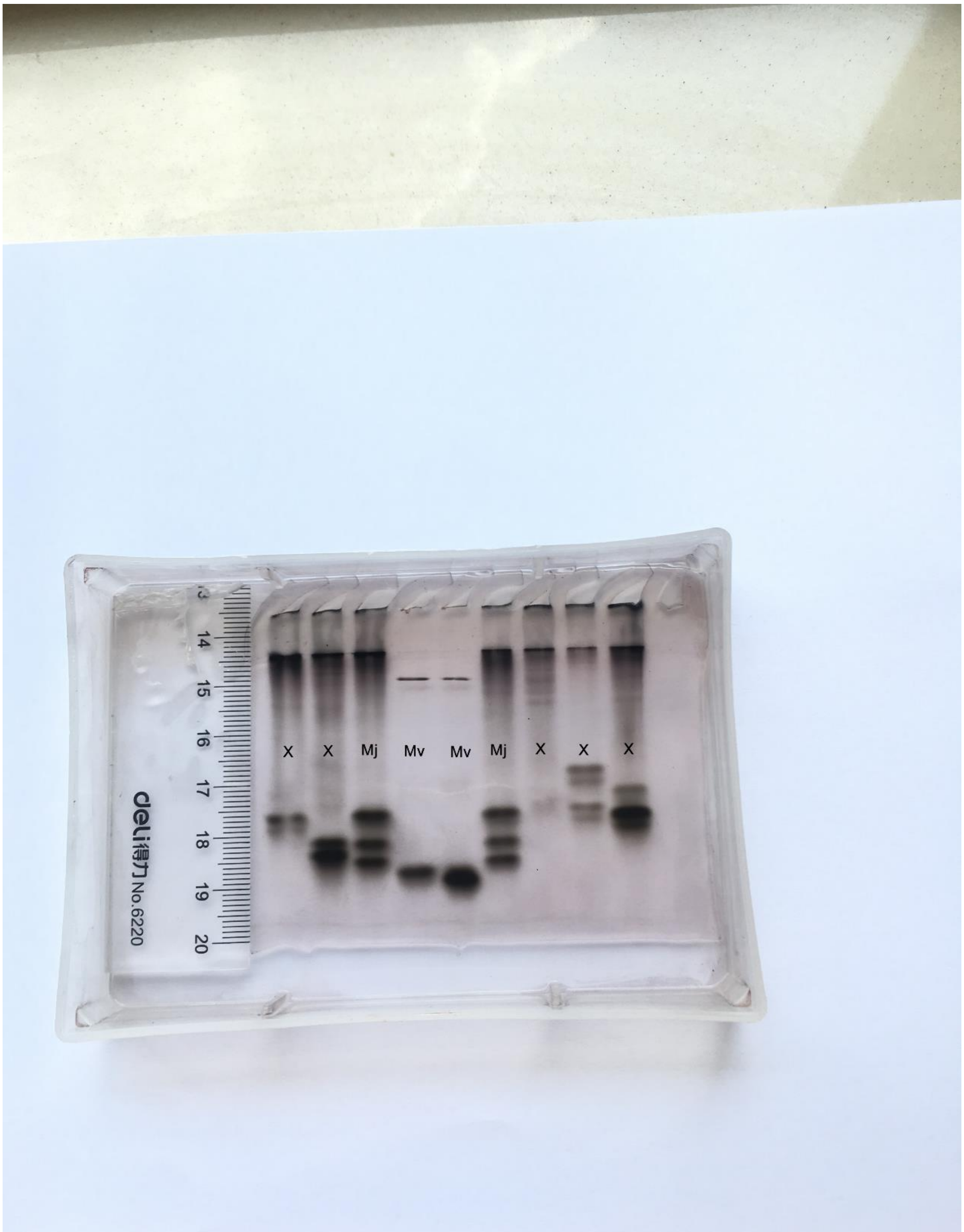

Fig 5-B: Esterase phenotype patterns obtained with electrophoresis of protein homogenates from five young egg-laying females of *Meloidogyne vitis* sp. nov. (lane Mv) and five young egg-laying females of the *Meloidogyne javanica* reference population (lane Mj). Lanes “X”: lanes not included in the final figure.

The image was captured from iPhone 6 A1699 (America).

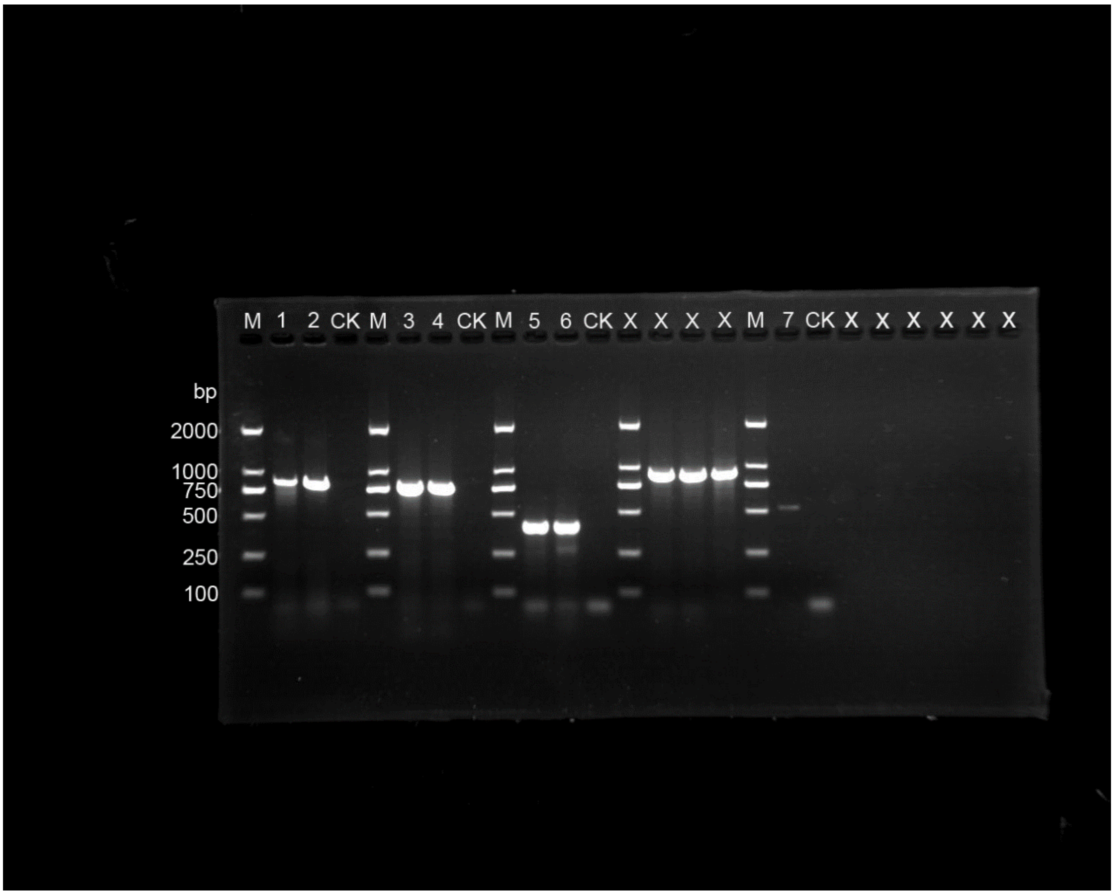

Fig 6 PCR electropherogram for different fragments of *Meloidogyne vitis* sp. nov. and *Meloidogyne mali*.

M: 2000 DNA marker; CK: The negative control consisting of water; Lanes 1-2: The ITS1-5.8S-ITS2 region of *Meloidogyne vitis* sp. nov. and *Meloidogyne mali,* respectively; Lanes 3-4: The D2/D3 region of *Meloidogyne vitis* sp. nov. and *Meloidogyne mali,* respectively; Lanes 5-6: The coxI region of *Meloidogyne vitis* sp. nov. and *Meloidogyne mali,* respectively; Lane 7: The coxII region of *Meloidogyne vitis* sp. nov. Lanes “X”: lanes not included in the final figure. The image was captured from LG2020 gel imaging system (Hang Zhou, China).

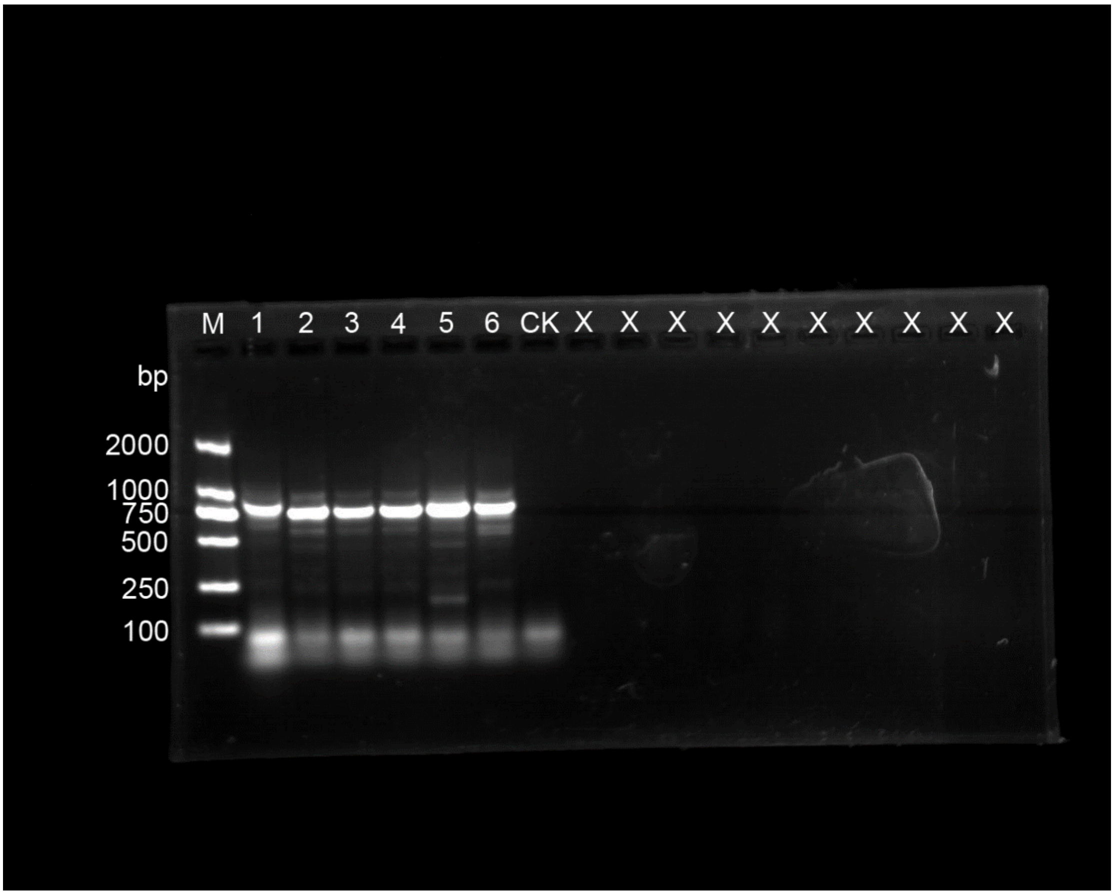

Fig 11-A: PCR amplification of the supplied RKNs with the Mv-F/Mv-R primers.

M: 2000 DNA marker; CK: The negative control consisting of water. Lanes 1-6: the ITS1-5.8S-ITS2 region of *Meloidogyne vitis* sp. nov., *Meloidogyne incognita*, *Meloidogyne javanica*, *Meloidogyne arenaria*, *Meloidogyne hapla* and *Meloidogyne enterolobii,* respectively. Lanes “X”: lanes not included in the final figure.

The image was captured from LG2020 gel imaging system (Hang Zhou, China).

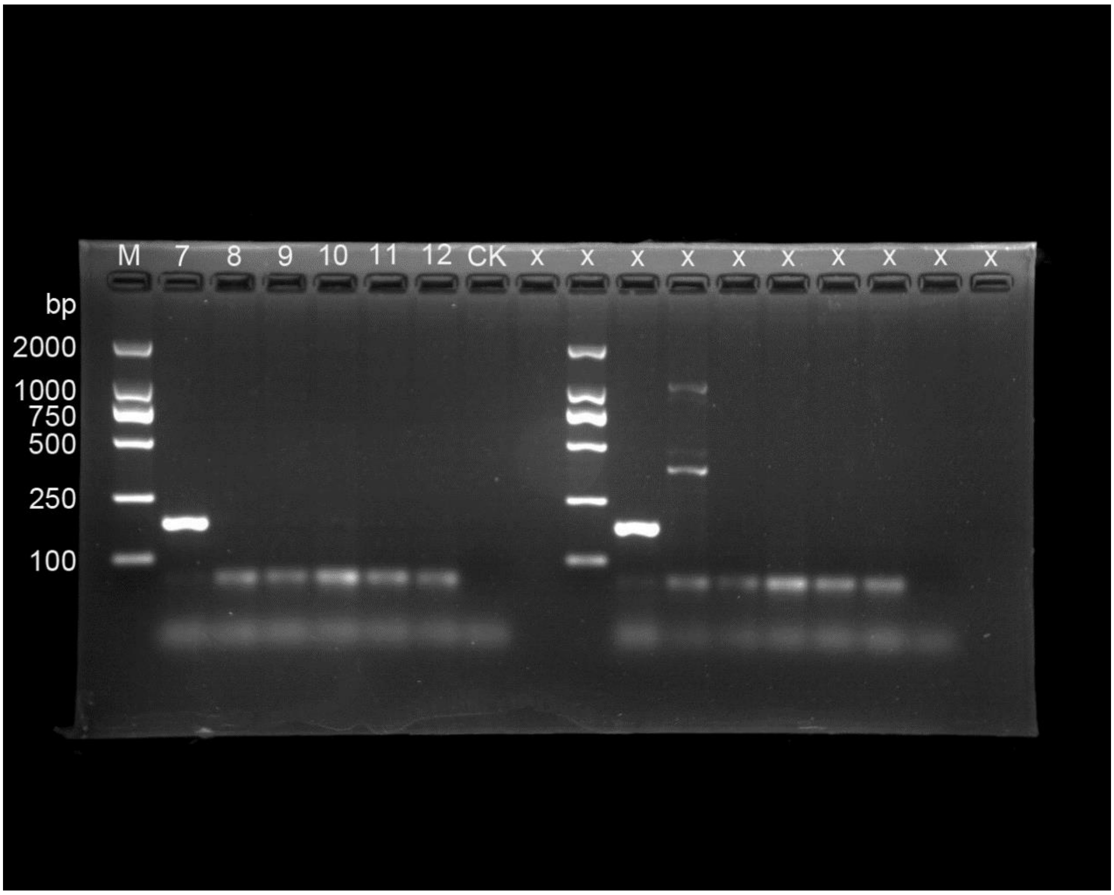

Fig 11-B: PCR amplification of the supplied RKNs with the Mv-F/Mv-R primers.
M: 2000 DNA marker; CK: The negative control consisting of water. Lanes 7-12: the amplification results of root-knot nematode species-specific PCRs of *Meloidogyne vitis* sp. nov., *Meloidogyne incognita*, *Meloidogyne javanica*, *Meloidogyne arenaria*, *Meloidogyne hapla* and *Meloidogyne enterolobii*, respectively. Lanes "X": lanes not included in the final figure.
The image was captured from LG2020 gel imaging system (Hang Zhou, China).
