# Supplementary material for: A new root-knot nematode, Meloidogyne vitis sp. nov. (Nematoda: Meloidogynidae), parasitizing grape in Yunnan
Source: PLoS One. 2021 Feb 3;16(2):e0245201. doi: 10.1371/journal.pone.0245201 (PMC7857618; doi:10.1371/journal.pone.0245201)
Supplement: Attachment — Submitted filename: Response to Reviewers.docx [file pone.0245201.s006.docx]

Dear editors and reviewers:

Thank you very much for reviewing our manuscript and giving us so much valuable guidance, We have modified all the problems pointed out in our manuscript, the details as follows:

**Response to the Reviewer #1:**

**The main problems pointed out by reviewer #1 and my corresponding modification as follows:**

**1. Look at lines 499-501, none of this is in the M&M section, all of that needs to be presented in the correct section.**

Answer: The method covered in lines 499-501 about the methods of outgroup taxa chosen and Phylogenetic trees construction has been removed and was stated in the materials and methods section. Besides, the same problem exists in Lines 450-451, 473-474, 522-523, all of these have been removed.

**2. Do not start sentences with *M. incognita*, it is *Meloidogyne incognita*.**

Answer: Lines 67 and 70 of manuscript both start sentences with “*M. incognita*”, and the “*M. incognita*” have been replaced with “*Meloidogyne incognita*”. Besides, the same problem exists in Lines 68, 338, 353, 358, 366, 369, 380, 387, 393, 397, 401, 455, 476, 478, 502, 504, 524, 636, they all have been modified.

**3. de Man indices (not formula)**

Answer: The word “formula” in the line 124 has been modified as “indices”.

**The following is the response to line comment of Reviewer #1
4. Line 52: cultivation has been conducted for thousands**

Answer: The sentence “cultivation has a history of thousands of years” in the line 52 has been modified as “cultivation has been conducted for thousands” .
**5. Line 58: Root-knot nematode infestation of grape has been documented in southern**

Answer: The sentence “Grape RKN disease has been discovered in southern” in the line 58 has been modified as “Root-knot nematode infestation of grape has been documented in southern”.
**6. Line 59: “fleshy”?, not really.**

Answer: The “peculiar fleshy roots are” in the line 59 has been modified as “root system are well developed and is”.
**7. Lines 60-61: only roots are infected, so not first, infection does not expand knots are induced**

Answer: After consideration, the sentence “The roots of grapes are usually infected first, and the infection expands to form a root knot” in lines 60-61 has been removed.
**8. Line 67: double citation Liu et al. (2018) [18], same with line 73**

Answer: The “[18]” contained in the sentence “Liu et al. (2017) [18] reported that grapes from the Huaihai economic zone were infected by *M. incognita*” of line 67 was moved to the end of the sentence. Same with line 73.
**9. Line 77: to further increase damage to grapes**

Answer: The “to destroy grapes” in the line 77 has been modified as “to further increase damage to grapes”.
**10. Lines: 87-97 remove, this is for materials and methods, and results sections**

Answer: the contents in lines 87-97 has been removed.
**11. Line 137: much more information about scanning electron microscope is needed.**

Answer: The instrument type and manufacturer of scanning electron microscope were added in the line 137.
**12. Line 140: Isozyme needs more detail**

Answer: Much more information about isozyme were added, mainly include sample preparation, electrophoretic procedure, gel stain, and a reference was added and listed in the reference section. Therefore, the order in which the reference appear in manuscript text and reference section were realignment.

**13. Line 207: severely nematode**

Answer: The “seriously nematode” in the line 207 has been modified as “severely nematode”.
**14. Line 212: Adult female heads were found associated with the xylem**

Answer: The sentence “The female adults could be seen biting tightly on the xylem when the cuticle was removed” in lines 212-213 has been modified as “Adult female heads were found associated with the xylem”.
**15. Line 356: DGO is used several times define, I am assuming this is the DEGO, dorsal esophageal gland opening.**

Answer: All the word “DGO” in this manuscript has been modified as “DEGO”.
**16. Line 408: define abbreviations**

Answer: The abbreviation “Mdh” and “Est” has been defined in “Isozyme phenotype electrophoresis” section of materials and methods.

**17. Lines 426-425: remove these are M&M**

Answer: After carefully reading, the contents of lines 423-425 (not 426-425) are M&M, so, the contents of lines 423-425 were removed and the following statements were slightly adjusted.
**18. Line 456: not in the M&M sections**

Answer: The sequence alignment method has been added to “Phylogenetic analyses” section of materials and methods, and slightly adjusted the related content; Besides, the same problem exists in lines 479, 505, 526, so, they were slightly adjusted as the same the line 456.

**19. Lines 577-579: Is it? I haven’t done it in 30 years**

Answer: The contents of lines 577-579 has been removed and the reference [48], [49] also have been removed, and slightly added some related contents. Therefore, the order in which the reference appear in manuscript text and reference section were realignment.
**20. Lines 625-633: repeating of M&M and results**

Answer: The contents of lines 625-633 has been removed.

**Response to Napsi Szincsak:**

**1. In the Methods section, include a sub-section called "Nomenclature".**

Answer: Root-knot nematode belong to invertebrate animal, therefore, the nomenclatural acts in my manuscript was carried out according to the guidelines of zoological names in Plos One website: <http://www.plosone.org/static/guidelines#botanical>. The detail content of nomenclatural acts was added into our manuscript.

**2. In the Results section, indicate where the globally unique identifier (GUID) will be added using XXXXX or an equivalent placeholder.**

Answer: We have indicated where the globally unique identifier (GUID) would be added using “XXXXXXX”.

Besides, How do I obtain the globally unique identifier (GUID) for the new species？I need your help, thank you very much！

**Response to** **Ulrich Melcher:**

**1. Please ensure that your manuscript meets PLOS ONE's style requirements, including those for file naming.**

Answer: We have main modified the reference of our manuscript according to PLOS ONE's style requirements in website:

https://journals.plos.org/plosone/s/file?id=wjVg/PLOSOne_formatting_sample_main_body.pdf

The main modifications are as follows: (a) the journal name was abbreviated. (b) the references [20] was cited as chapter in a book, so, it was modified according to the guidelines for chapter in a book references of PLOS ONE's style requirements. (c) use the symbol “[ ]” to enclose the title of chinese reference. (d) the references [25], [26], [51] was cited as book, so, it was modified according to the guidelines for book references of PLOS ONE's style requirements. (e) the references [39] was cited as Masters' theses, so, it was modified according to the guidelines for Masters' theses references of PLOS ONE's style requirements.

**2. The problem about data Availability statement**

Answer: All sequences are available from the GenBank database (accession number MN816222, MN816223, MN816224, MN816225, MN816226, MN814829, MN814830, MN814831, MT012386 and MT012387). Other relevant data are within the manuscript and its Supporting Information files.

**3. The problem about original uncropped and unadjusted images**

Answer: The original uncropped and unadjusted images of our manuscript have been submitted as supporting information with PDF file, the file name is 'S1_raw_images'.

**4. PLOS requires an ORCID iD for the corresponding author**

Answer: I have registered an ORCID iD and updated my Information in PLOS ONE editorial manager submission system.

**5. The problem that using the Preflight Analysis and Conversion Engine (PACE) digital diagnostic tool to deal with image.**

Answer: The figure files have been modified using the Preflight Analysis and Conversion Engine (PACE) digital diagnostic tool except the Supporting Information files.

We appreaited the valuable comments from the reviewers and the editors. Thank you very much for your continued attention.

Sincerely

Xianqi Hu

Professor

College of Plant Protection

Yunnan Agricultural University

Kunming 650201, Yunnan, China

[xqh@ynau.edu.cn](mailto:hxq@ynau.edu.cn)
