# Supplementary material for: A new root-knot nematode, Meloidogyne vitis sp. nov. (Nematoda: Meloidogynidae), parasitizing grape in Yunnan
Source: PLoS One. 2021 Feb 3;16(2):e0245201. doi: 10.1371/journal.pone.0245201 (PMC7857618; doi:10.1371/journal.pone.0245201)
Supplement: Attachment — Submitted filename: Response to Reviewers.docx [file pone.0245201.s007.docx]

Dear editors and reviewers:

Thank you very much for reviewing our manuscript and giving us so much valuable guidance, we have modified all the problems pointed out in our manuscript, the details as follows:

**Response to the Additional Editor:**

**The main problems pointed out by additional editor and my corresponding modification as follows:**

**In abstract：**

**1. …pore is located on ventrally region -->pore is located on the ventral region**

Answer: The sentence “pore is located on ventrally region” in the line 21 has been modified as “pore is located on the ventral region”.

**2. I don’t understand the difference between “not smooth{ and :slightly wrinkled”**

Answer: The words “not smooth” and “slightly wrinkled” in the sentence “Second-stage juveniles are characterized by a head region that is not smooth and slightly wrinkled” both mean the head region is not smooth and they have no obvious difference. After consideration, the sentence “Second-stage juveniles are characterized by a head region that is not smooth and slightly wrinkled” in lines 27-28 has been modified as “Second-stage juveniles are characterized by a head region with slightly wrinkled mark”.

**3. …The new species has rare Mdh (N3d) and Est phenotypes (VF1).**

Answer: The sentence “The new species has a rare Mdh phenotype (N3d) and Est phenotype (VF1)” in the line 31 has been modified as “The new species has rare Mdh (N3d) and Est phenotypes (VF1)” .

**4. “Collected M. mali and amplified the sequences as mentioned above and compared with the corresponding sequence of new species, the result showed that all of these sequences were significantly different.” Different from what?**
Answer: The sentence “Collected *M.mali* and amplified the sequences as mentioned above and compared with the corresponding sequence of new species, the result showed that all of these sequences were significantly different” in lines 34-36 is want to express that there are highly base divergence between the new species and *M.mali* in the sequences as mentioned above, therefore, this sentence has been modified as “*Meloidogyne mali* was collected for amplifying these sequences as mentioned above, which were compared with the corresponding sequences of new species, the result showed that all of these sequences with highly base divergence (48-210 base divergence)” .
**5. Second-stage juveniles are characterized by a head region that is not smooth and slightly wrinkled, a labial disc fused with the medial lips to form a dumbbell-shaped structure, a slightly sunken into the middle of the medial lips**

Answer: The sentence “Second-stage juveniles are characterized by a head region that is not smooth and slightly wrinkled, a labial disc fused with the medial lips to form a dumbbell-shaped structure, a slightly sunken into the middle of the medial lips” in lines 27-29 has been modified as “Second-stage juveniles are characterized by a head region with slightly wrinkled mark, a labial disc fused with the medial lips to form a dumbbell-shaped structure, a slightly sunken appearance of the middle of the medial lips”.
**6. SCAR?**

Answer: The word “SCAR” is the abbreviations of “sequence characterized amplified region”. Specific primers have been developed to PCR-amplify diagnostic repetitive regions of sequence: sequence characterized amplified region (SCAR). SCAR primers are designed based on random amplified polymorphic DNA (RAPD) product and rDNA sequences and are preferred for identification purposes as the relatively high annealing temperatures that are used with species-specific primers enhance their specificity. After consideration, the sentence “SCAR primers were designed” has been modified as “sequence characterized amplified region (SCAR) primers for rapid identification of this new species were designed”.

**Response to** **Ulrich Melcher:**

**1. The problem about financial disclosure**

Answer: The financial disclosure of our manuscript do not make changes.

**2. The problem about laboratory protocols**

Answer: We have no laboratory protocols.

**3. The problem that using the Preflight Analysis and Conversion Engine (PACE) digital diagnostic tool to deal with image.**

Answer: The figure files have been modified using the Preflight Analysis and Conversion Engine (PACE) digital diagnostic tool except the Supporting Information files.

We appreaited the valuable comments from the reviewers and the editors. Thank you very much for your continued attention.

Sincerely

Xianqi Hu

Professor

College of Plant Protection

Yunnan Agricultural University

Kunming 650201, Yunnan, China

[xqh@ynau.edu.cn](mailto:hxq@ynau.edu.cn)
